# A regularly irregular rhythm—what is the diagnosis?

**DOI:** 10.1007/s12471-015-0789-z

**Published:** 2016-01-04

**Authors:** A.-D. Margulescu, O.A. Enescu, D. Vinereanu

**Affiliations:** 1 University of Medicine and Pharmacy Carol Davila, Bucharest, Romania; 2 Department of Cardiology, University and Emergency Hospital of Bucharest, Bucharest, Romania

## Answer

**Fig. 1:**
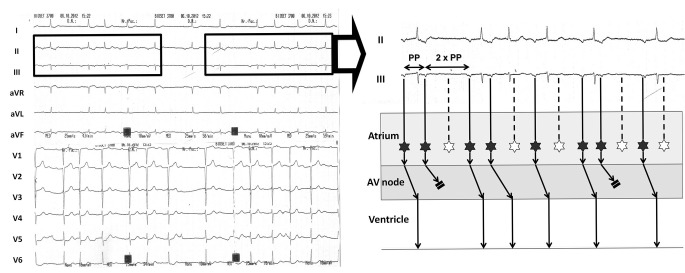
*Left*: Twelve-lead ECG at admission. The rhythm is regularly irregular, and there is a repetitive pattern in the rhythm (boxes); this suggests the presence of Wenckebach phenomena. *Right*: Ladder diagram depicting the mechanism of the arrhythmia. For simplicity, only leads II and III from the boxes on the left are displayed. The atrial rhythm is ectopic (P waves are negative in inferior leads) and irregular, with the longer PP interval twice the shorter PP interval. The atrial ectopic rate is 130 beats/min; hence, this is atrial tachycardia (originating from the inferior aspect of the atria) with variable Mobitz type II exit block to the atrium (in the ladder diagram, black stars denote atrial ectopic beats; white stars denote ectopic beats with exit block to the atrium). Furthermore, the atrial tachycardia is conducted to the ventricle with progressively longer PR intervals until some P waves are blocked. This behaviour is typical of Wenckebach phenomena in the atrioventricular (AV) node

The ECG on admission shows ectopic atrial tachycardia with multilevel block: variable Mobitz type II exit block to the atrium and Wenckebach phenomena in the atrioventricular node (see Fig. 1 for detailed explanation).

This complex arrhythmia was highly unusual, and suggested a cardiac infiltrative process. Transthoracic echocardiogram showed normal cardiac function, but the interatrial septum and both atrial roofs were abnormally thick (not shown). Chest computed tomography scan also showed mediastinal adenopathy (not shown). This combination of findings narrowed the differential diagnosis to atrial infiltration due to sarcoidosis or lymphoma/tumours. Biopsies collected during minimal thoracotomy established the diagnosis of sarcoidosis (not shown).

In cardiac sarcoidosis, conduction disturbances are related to granulomatous infiltration of the conduction system [1]. Their evolution is unpredictable and device therapy (either permanent pacing or cardiac defibrillator implantation) is recommended [2]. The patient initially declined device implantation. Prednisolone was started but had to be discontinued 7 months later due to development of Cushing’s syndrome. Eighteen months after diagnosis the patient was readmitted with complete heart block and consented to dual-chamber pacemaker implantation. One year afterwards her ventricular function remained normal; interrogation of the device disclosed no ventricular arrhythmias at follow-up.

This case underlines that when conduction disturbances are seen in younger patients, a search for cardiac structural abnormalities is warranted and sarcoidosis should be suspected [3].
